# Systemic RALA/iNOS Nanoparticles: A Potent Gene Therapy for Metastatic Breast Cancer Coupled as a Biomarker of Treatment

**DOI:** 10.1016/j.omtn.2016.12.010

**Published:** 2016-12-31

**Authors:** Cian M. McCrudden, John W. McBride, Joanne McCaffrey, Ahlam A. Ali, Nicholas J. Dunne, Vicky L. Kett, Jonathan A. Coulter, Tracy Robson, Helen O. McCarthy

**Affiliations:** 1 School of Pharmacy, Queen’s University Belfast, 97 Lisburn Road, Belfast BT9 7BL, Northern Ireland; 2 Department of Pharmacology and Therapeutics, University College Cork, Cork T12 YN60, Ireland; 3 School of Mechanical and Manufacturing Engineering, Dublin City University, Dublin 9, Ireland; 4 Royal College of Surgeons in Ireland, 123 St. Stephen’s Green, Dublin 2, Ireland

**Keywords:** nonviral gene therapy, nitric oxide, nanoparticle, breast cancer, metastasis

## Abstract

This study aimed to determine the therapeutic benefit of a nanoparticular formulation for the delivery of inducible nitric oxide synthase (*iNOS*) gene therapy in a model of breast cancer metastasis. Nanoparticles comprising a cationic peptide vector, RALA, and plasmid DNA were formulated and characterized using a range of physiochemical analyses. Nanoparticles complexed using *iNOS* plasmids and RALA approximated 60 nm in diameter with a charge of 25 mV. A vector neutralization assay, performed to determine the immunogenicity of nanoparticles in immunocompetent C57BL/6 mice, revealed that no vector neutralization was evident. Nanoparticles harboring *iNOS* plasmids (constitutively active cytomegalovirus [CMV]-driven or transcriptionally regulated human osteocalcin [hOC]-driven) evoked *iNOS* protein expression and nitrite accumulation and impaired clonogenicity in the highly aggressive MDA-MB-231 human breast cancer model. Micrometastases of MDA-MB-231-luc-D3H1 cells were established in female BALB/c SCID mice by intracardiac delivery. Nanoparticulate RALA/CMV-*iNOS* or RALA/hOC-*iNOS* increased median survival in mice bearing micrometastases by 27% compared with controls and also provoked elevated blood nitrite levels. Additionally, *iNOS* gene therapy sensitized MDA-MB-231-luc-D3H1 tumors to docetaxel treatment. Studies demonstrated that systemically delivered RALA-*iNOS* nanoparticles have therapeutic potential for the treatment of metastatic breast cancer. Furthermore, detection of nitrite levels in the blood serves as a reliable biomarker of treatment.

## Introduction

An obstacle to genetic therapies is the absence of a vector with the DNA delivery ability of a virus that lacks the immunogenicity commonly associated with viral vectors. We have developed a cationic fusogenic peptide vector, RALA, that, on exposure to anionic nucleic acids, self-assembles into nanoscale particles suitable for cell membrane penetration. Endosomal escape, consequent to conformational change at low pH, ensures that the genetic cargo can reach the nucleus and achieve transgene expression.^1^ We previously demonstrated the remedial potential of RALA-delivered therapeutic cargoes. Growth of ZR-75-1 breast cancer xenografts was abrogated by plasmid FK506-binding protein-like (*FKBPL*),^2^ whereas nanocomplexation of anionic bisphosphonates with RALA afforded the agents cytotoxicity against PC-3 prostate cancer cells in vitro and in xenografts following intratumoral injection.^3^ In this study, we aimed to provoke a therapeutic benefit in a model of aggressive breast cancer by nanocomplexation of plasmid inducible nitric oxide synthase (*iNOS*) with RALA.

The paradoxical relationship between nitric oxide (·NO) and transformed tissue, whereby low concentrations of the gasotransmitter provoke an aggressive phenotype but higher concentrations are detrimental to the tumor,^4^ has led to a divergence in the discipline, with attempts being made to either promote or interfere with ·NO signaling. The mechanisms by which ·NO mediates its effects in neoplastic conditions are diverse but can be broadly characterized into promotion (low ·NO) or inhibition (high ·NO) of apoptosis, promotion (low) or inhibition (high) of proliferation, and stimulation (low) or attenuation (high) of angiogenesis.^4^ ·NO can react with inorganic molecules (i.e., oxygen, superoxide, or transition metals), structures in DNA, prosthetic groups, or proteins and can elicit beneficial or detrimental responses dependent on radical concentration and local environmental conditions.^5^ Host macrophages that infiltrate tumors rely partially on the cytotoxic properties of ·NO to evoke an anti-tumoral response.^6^

The majority of attempts to exploit the tumoricidal properties of ·NO involve using an ·NO donor molecule. Many such donors exist and are broadly represented by the organic nitrates, metal-NO complexes, *S*-nitrosothiols, sydnonimines, diazeniumdiolates (NONOates), and ·NO-drug hybrids.^4^ One ·NO-donating prodrug that has received particular attention is JS-K. JS-K induced apoptosis in a range of breast cancer cell lines but spared normal human microvascular endothelial cells (HMECs) and MCF-10A.^7^ JS-K was recruited into the National Cancer Institute’s Rapid Access to Interventional Development (RAID) program, accelerating its progression as a potential therapeutic agent.^8^

As an alternative approach to achieving therapeutic levels of intra-tumoral ·NO, we,^9^, ^10^, ^11^, ^12^, ^13^, ^14^ and others^15^, ^16^, ^17^ have demonstrated the benefit of *iNOS* as a therapeutic transgene. Constitutive *iNOS* expression abolished clonogenicity in ZR-75-1 breast cancer cells^13^ and sensitized to cisplatin in human cancer cell lines and murine RIF-1 xenografts^9^ and in A549 models of human primary and metastatic lung cancer.^17^ To limit ·NO release from an *iNOS* gene therapeutic to target tumors, we have deployed a transcriptional targeting approach using the human osteocalcin (hOC) promoter to drive *iNOS* expression. The hOC promoter is activated by transcription factors such as *Runx2* and *Fra-2*, which are commonly overexpressed in cancers that metastasize to bone.^18^ hOC-*iNOS*-derived ·NO achieved almost complete elimination of colony-forming ability in PC-3 and DU145 castration-resistant prostate cancer cells and induced stasis in PC-3 xenografts.^11^, ^19^

The purpose of the current study was to determine whether cationic RALA-based nanoparticles (NPs) carrying an *iNOS* transgene had a therapeutic effect in mice bearing MDA-MB-231 (known to be sensitive to the ·NO donor diethylenetriamine (DETA)/NO through generation of dinitrogen trioxide)^20^ micrometastases.

## Results

### Nanoparticle Characterization

**Figure 1:**
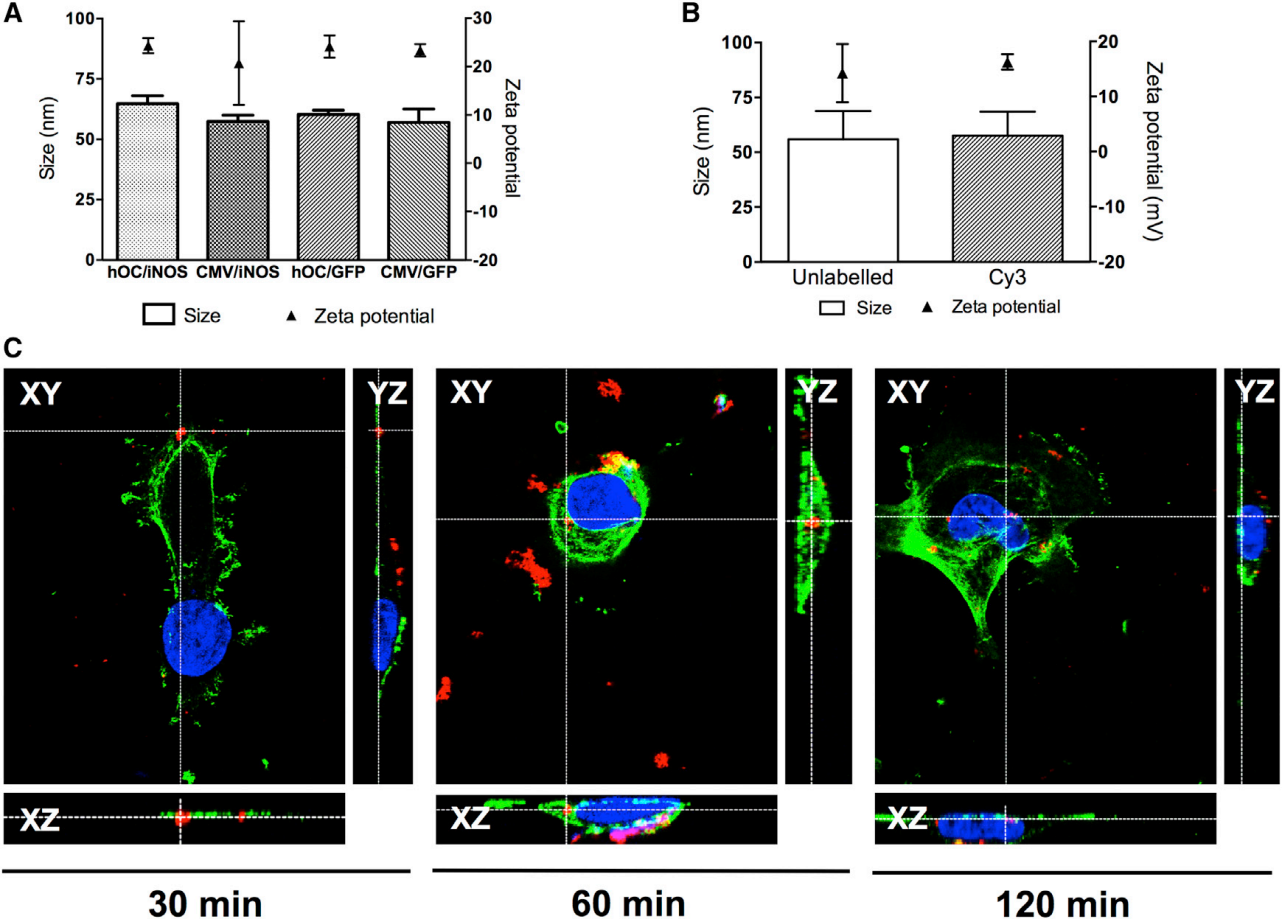
Complexation of Plasmid DNA with RALA Produces Nanoparticles Suitable for Cellular Delivery (A) Incubation of plasmid DNA with RALA resulted in nanoparticles that did not exceed 100 nm in diameter, with a positive charge of approximately 20–25 mV. (B) Cy3-labeled DNA forms nanoparticles with RALA that resemble those formed with unlabeled DNA. Data points represent mean ± SD. n ≥ 3. (C) Orthogonal sectioning of z stacks of MDA-MB-231-luc-D3H1 cells transfected with RALA/Cy3-pEGFP-1. RALA delivers plasmid DNA to the nuclei of MDA-MB-231-luc-D3H1 cells within 120 min. Green, actin cytoskeleton; blue, nucleus; red, Cy3.

Incubation of RALA with plasmid DNA in water resulted in the formation of nanoparticles with physical characteristics suitable for cellular internalization (Figure 1A).^1^, ^2^, ^21^

### Subcellular Nanoparticle Localization

Labeling with Cy3 did not affect the physicochemical properties of nanoparticles (Figure 1B). The ability of RALA to deliver Cy3-labeled pEGFP-1 nanoparticles to the nuclei of MDA-MB-231-luc-D3H1 cells was confirmed by confocal fluorescence microscopy using orthogonal sectioning (to construct XZ and YZ images to correspond to an area of interest in an XY image following collection of a z stack of images). By 60 min following commencement of transfection, Cy3 fluorescence was evident within the confines of the cell and, within 120 min, was detected within the nucleus (Figure 1C).

### Vector Neutralization Assay

**Figure 2:**
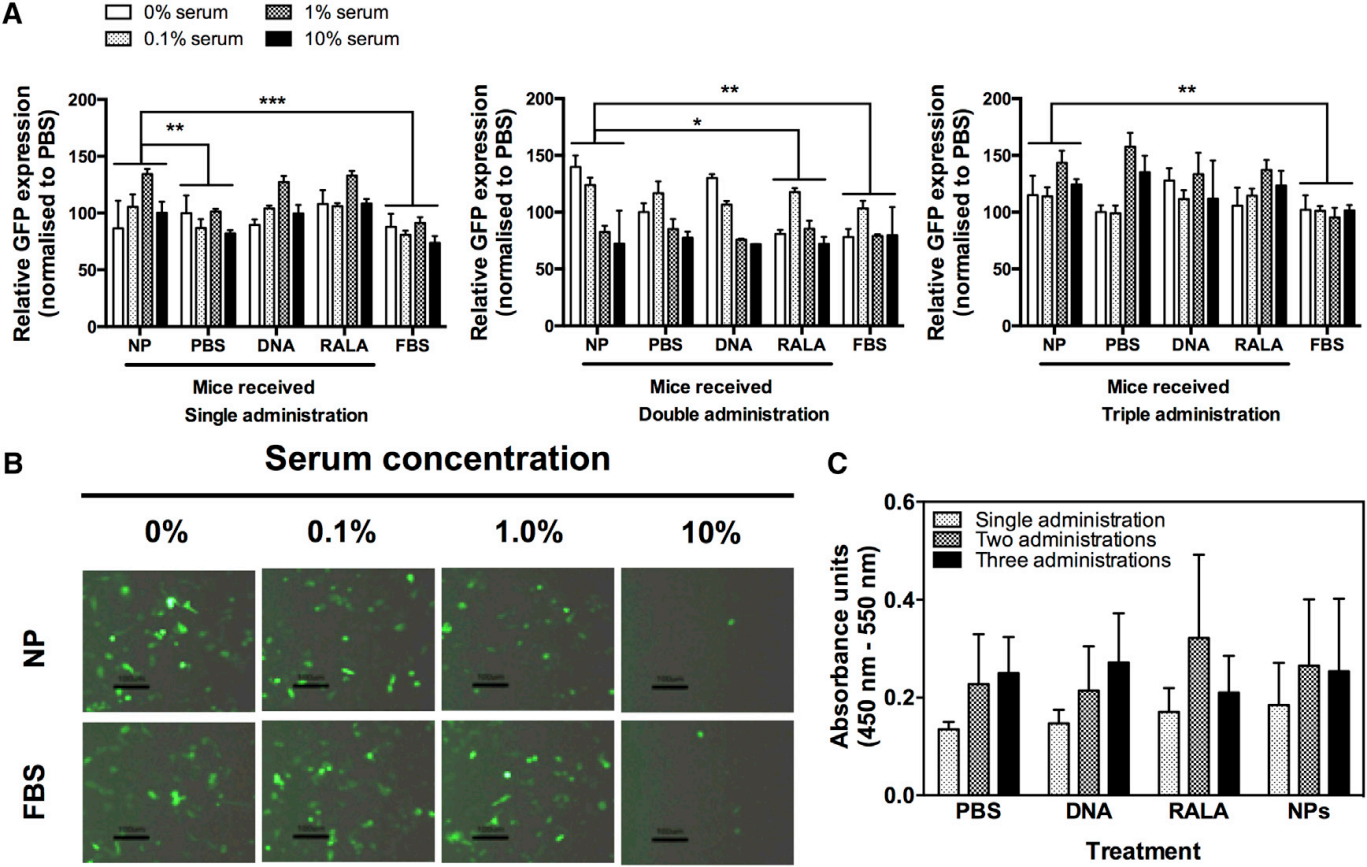
Administration of RALA/pEGFP-N1 Nanoparticles to Immunocompetent Mice Does Not Provoke a Neutralizing Antibody Response (A) Flow cytometric analysis of *GFP* in ZR-75-1 cells after incubation of RALA/pEGFP-N1 nanoparticles with sera from C57BL/6 mice that received the indicated treatment (PBS/DNA/RALA/NPs) weekly for up to 3 weeks. *p < 0.05, **p < 0.01, ***p < 0.001 compared with expression elicited by RALA/pEGFP-N1 NPs that had been incubated in sera from mice that had received nanoparticles (multiple comparisons ANOVA). (B) Fluorescence micrographs of ZR-75-1 cells transfected with RALA/pEGFP-N1 nanoparticles following incubation in FBS or sera from mice that received two administrations of RALA/pEGFP-N1. (C) Serum-neutralizing antibody (immunoreactivity of an anti-mouse IgA, IgG, and IgM) content analyzed by ELISA. Data points represent mean ± SD, n ≥ 3.

The transfection potency of RALA/pEGFP-N1 in ZR-75-1 breast cancer cells was not detrimentally affected by incubation of the nanoparticles with pooled sera from mice that had received RALA/pEGFP-N1 nanoparticles (single or multiple administrations thereof). Repeated measures two-way ANOVA with Dunnett’s correction for multiple comparisons was used to compare sera from nanoparticle-treated mice with other treatments (Figure 2A). In no case did incubation in sera from nanoparticle-treated mice lessen *GFP* expression; rather, nanoparticles incubated in sera from nanoparticle-treated mice provoked a slightly higher transfection ability. The degree of fluorescence of ZR-75-1 was diminished slightly when nanoparticles were incubated in 10% serum, although this cannot be due to antibody neutralization because nanoparticles incubated in sera from mice that received PBS, plasmid DNA (pDNA), or RALA only, or those incubated in fetal bovine serum (FBS), also evoked less fluorescence when the serum concentration was 10% (Figure 2B).

Sera from mice that received PBS, pEGFP-N1, RALA, or RALA/pEGFP-N1 nanoparticles produced limited immunoreactivity in RALA/pEGFP-N1 nanoparticle-coated wells of an ELISA plate (Figure 2C). There was no significant difference (p > 0.05) in immunoreactivity between sera from mice that received nanoparticles and mice that received any other treatment (repeated measures two-way ANOVA with Tukey multiple comparisons test).

### *iNOS* Transgene Expression in MDA-MB-231-luc-D3H1 Cells

**Figure 3:**
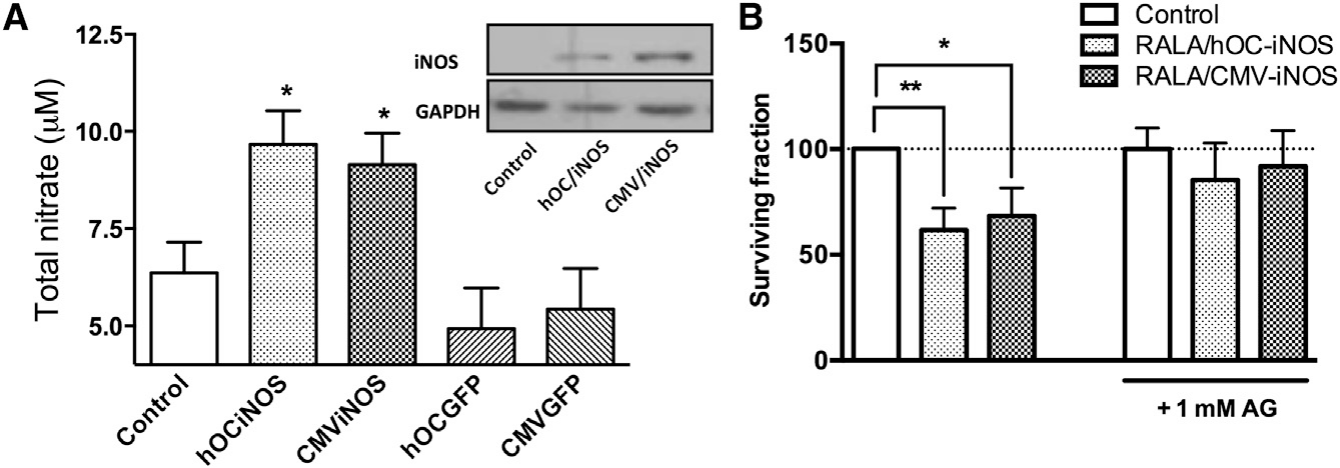
Validation of hOC- and CMV-Driven *iNOS* Plasmids (A) iNOS protein expression in MDA-MB-231-luc-D3H1 cells 48 hr post-transfection with RALA/hOC-*iNOS* or RALA/CMV-*iNOS* (comprising 0.5 μg DNA) at N:P10 for 6 h. ·NO generation was confirmed by Greiss test. (B) MDA-MB-231-luc-D3H1 cells overexpressing *iNOS* form fewer clonogenic colonies, which is partially inhibited by 1 mM aminoguanidine. Data points represent mean ± SD, n = 3.

Transfection of MDA-MB-231-luc-D3H1 cells with cytomegalovirus (CMV)- or hOC-*iNOS* provoked accumulation of nitrites in the culture medium, as analyzed 48 hr post transfection; iNOS protein expression was also detectable by western blot (Figure 3A).

### Clonogenics

MDA-MB-231-luc-D3H1 cells transfected with RALA/hOC-*iNOS* (61.70% ± 10.39) or RALA/CMV-*iNOS* (68.40% ± 13.32) had lower clonogenicity than untransfected control cells. Treatment with 1 mM aminoguanidine (a NOS inhibitor)^22^ partially blocked this inhibition of clonogenicity (79.4% ± 16.2 and 85.4% ± 15.6% of control for RALA/hOC-*iNOS* and RALA/CMV-*iNOS*, respectively) (Figure 3B). Optimization of transfection conditions is summarized in Figure S1.

### RALA/*iNOS* Gene Therapy Slows Progression of Metastatic Breast Cancer in Mice

**Figure 4:**
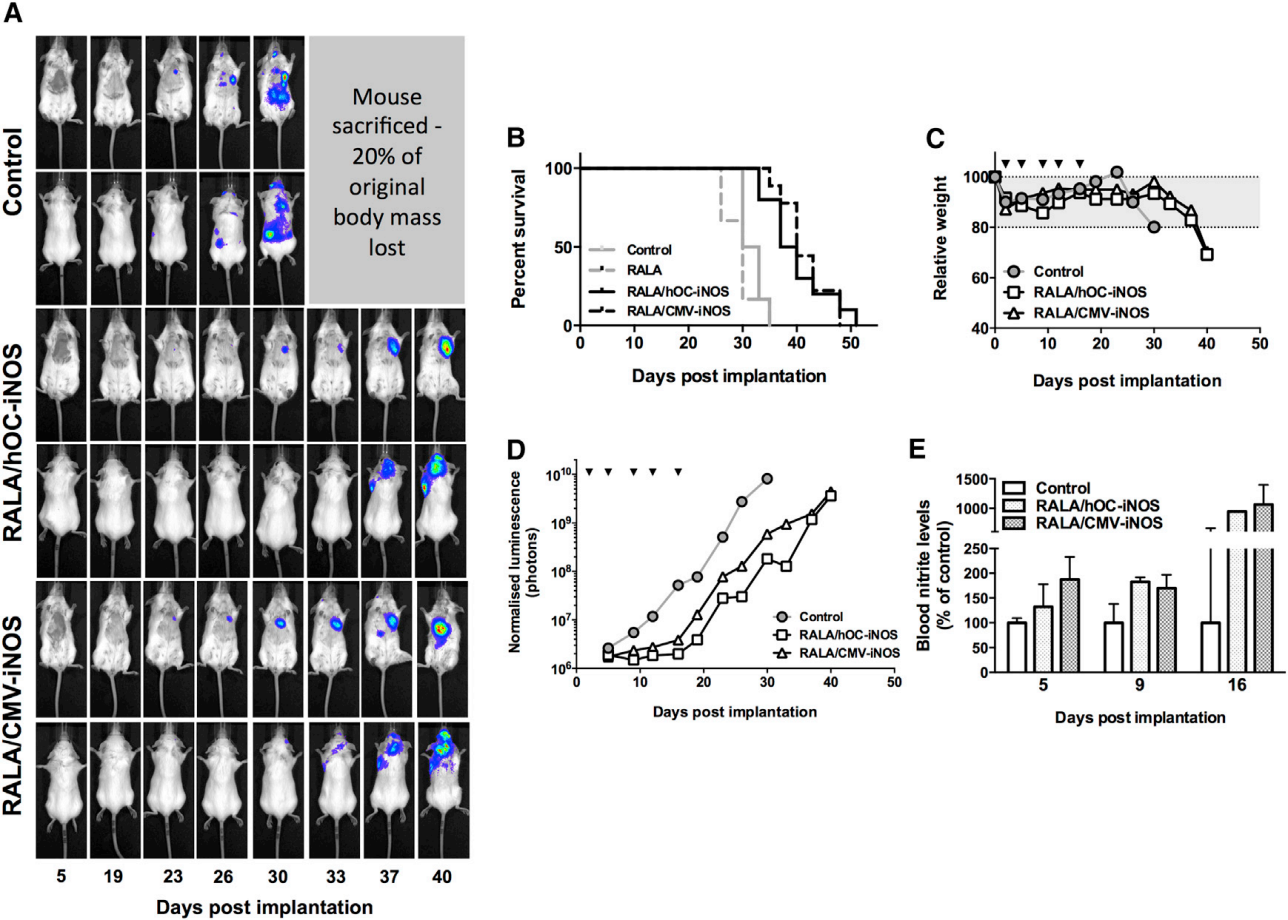
Treatment with RALA/hOC-*iNOS* or RALA/CMV-*iNOS* Improves the Survival of MDA-MB-231-luc-D3H1 Metastasis-Bearing Mice (A) IVIS images of mice (control and RALA/*iNOS*) at the indicated time points post-implantation. (B) Survival of metastasis-bearing mice (n = 6 [control, RALA] or ≥ 9 [either RALA/*iNOS* strategy]). (C) Weight loss of exemplary mice. (D) Total bioluminescence in exemplary mice. Inverted triangles denote treatment time points. (E) Relative blood nitrite levels in control, RALA/hOC-*iNOS*-, and RALA/CMV-*iNOS*-treated mice. Datapoints represent mean, and, where appropriate, error bars represent mean ± SD.

Administration of hOC/CMV-*iNOS*-loaded RALA nanoparticles delayed bioluminescence accumulation (Figures 4A and 4D) and disease progression in mice bearing MDA-MB-231-luc-D3H1 micrometastases (Figure 4B). Control and vehicle-only mice had a median post-implantation survival of 31.5 and 30.0 days, respectively. Median survival was significantly increased (log-rank [Mantel-Cox] test) by treatment with RALA/hOC-*iNOS* (38.5 days, p = 0.001) and RALA/CMV-*iNOS* therapy (40 days, p > 0.001).

Figures 4C and 4D comprise biochemical and physical data from a single mouse per treatment group (individuals whose post-implantation survival was closest to the relevant treatment’s median value; cumulative data of all mice are presented in Figures S2 and S3). Mice that received *iNOS* transgenes lost mass (Figure 4C) and developed a bioluminescent signal (Figure 4D) more slowly than the control.

Blood nitrite levels in both RALA/*iNOS* complex-receiving mice were up to 9-fold higher than the blood nitrite levels of control mice (Figure 4E). Opsonization and sequestration by the mononuclear phagocyte system is a common fate of cationic nanoparticles following systemic administration—this could explain why gene expression following treatment with RALA/p*Luciferase*^1^ and other similarly charged gene therapy nanoparticles^23^ is seen mainly in the lungs and livers of mice. To determine whether these organs were less susceptible to metastasis colonization in RALA/*iNOS*-treated mice, we attempted to quantify the number of metastatic lesions in mice at the endpoint and to make an estimation of the location of the lesions. The number of lesions evident in the final images (i.e., experimental endpoint) of each mouse was counted, their location was assigned as head, thoracic, abdominal, or skeletal, and the number at each location was counted. Mice that received *iNOS* gene therapy had fewer metastatic foci than control mice, and RALA/CMV-*iNOS* or RALA/hOC-*iNOS* treatment appeared to inhibit metastasis development in the abdominal cavity and the head but had no effect on lesion development in the skeleton or thoracic cavity (Figure S4). The inhibition of lesion development in the abdomen may be due to *iNOS* gene overexpression in the liver, although we did not investigate this further.

### *iNOS* Sensitizes to Docetaxel In Vitro and In Vivo

**Figure 5:**
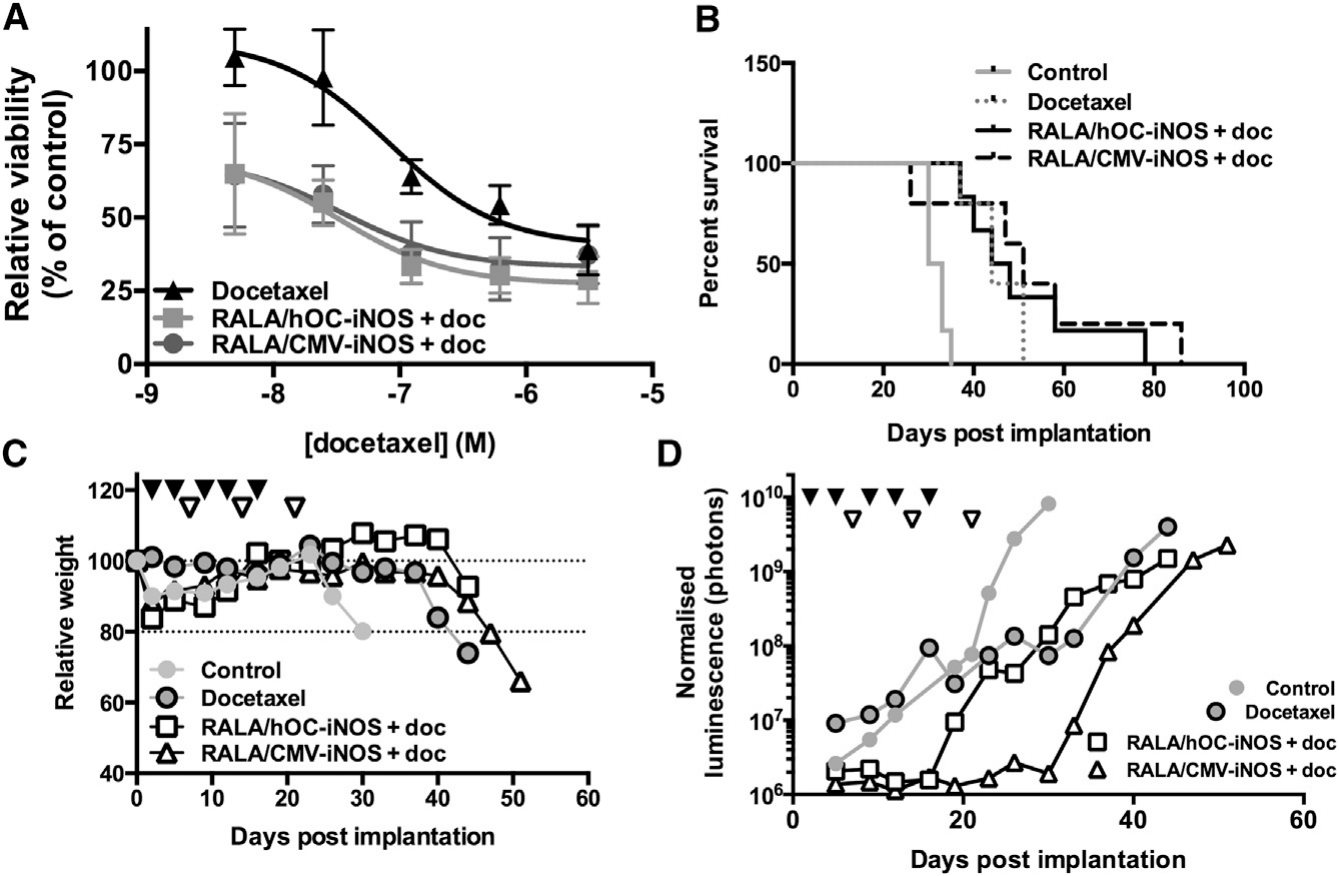
Assessment of *iNOS* Overexpression on Sensitization of MDA-MB-231-luc-D3H1 Cells to Docetaxel (A) Transfection of MDA-MB-231-luc-D3H1 cells with RALA/hOC-*iNOS* or RALA/CMV-*iNOS* increases the potency of docetaxel in vitro. (B) RALA/hOC-*iNOS* or RALA/CMV-*iNOS* treatment produces a slight additive improvement in response to docetaxel in MDA-MB-231-luc-D3H1 metastasis-bearing mice (n ≥ 5). (C and D) Weight loss and bioluminescence accumulation data for exemplary mice. Closed inverted triangles denote gene therapy treatment, and open inverted triangles denote docetaxel treatment. Datapoints represent mean, and, where appropriate, error bars represent mean ± SD.

Transfection of MDA-MB-231-luc-D3H1 cells with either RALA/CMV-*iNOS* or RALA/hOC-*iNOS* nanoparticle complexes before treatment with docetaxel enhanced the docetaxel response. Docetaxel dose-dependently inhibited the viability of MDA-MB-231-luc-D3H1 cells (effective concentration 50 [EC_50_] of 82.7 nM), whereas transfection with RALA/hOC-*iNOS* or RALA/CMV-*iNOS* reduced the EC_50_ to 33.3 nM and 34.9 nM, respectively (both p < 0.05, as assessed by repeated measures one-way ANOVA with Geisser-Greenhouse correction; Figure 5A).

Metastasis-bearing mice that were treated with docetaxel had a median survival of 44 days. Although co-administration of docetaxel with RALA/hOC-*iNOS* (46 days, p = 0.8601) or RALA/CMV-*iNOS* (49 days, p = 0.3757) complexes did not significantly improve median survival, maximal survival (51 days in docetaxel only) was considerably longer in both the docetaxel + RALA/hOC-*iNOS* and RALA/CMV-*iNOS* treatment groups (78 and 86 days, respectively; Figure 5B). Figures 5C and 5D represent mass loss and bioluminescence accumulation in individual mice whose survival was closest to median survival; cumulative data on all mice are presented in Figure S5. As is evident in Figure 5D, luminescence accumulation was retarded in the gene therapy plus docetaxel groups until therapy was withdrawn, whereas, in docetaxel-treated mice, luminescence accumulation progressed from day 5 onward, although at a slower rate than in the control.

## Discussion

The evidence presented here demonstrates, for the first time, the therapeutic utility of *iNOS* gene therapy following systemic administration. In our assays, both RALA/*iNOS* strategies impressively prolonged the survival of mice bearing MDA-MB-231 micrometastases. Using blood nitrite measurements, we demonstrated that receiving either gene therapy regimen provoked ·NO generation in these mice. Assessment of circulating nitrite concentrations in this system was a viable biomarker for successful transgene expression. Changes in ·NO flux have been used previously to confirm therapeutic ·NO generation,^24^ although this was by invasive insertion of an amiNO 700 probe.

Nanoparticles formed of RALA and either *iNOS* plasmid displayed size and charge characteristics suitable for cellular internalization. Indeed, our observations were in agreement with previous studies on the internalization of RALA/plasmid DNA nanoparticles, which occurs rapidly and relies on both clathrin- and caveolin-dependent processes.^1^ We have demonstrated previously that *iNOS* gene therapies delivered intratumorally produce an impressive therapeutic benefit^9^, ^10^, ^11^, ^12^, ^19^ and described reporter gene expression when the *Luciferase* gene was delivered systemically using RALA,^1^ but this is the first description of systemic RALA-mediated therapeutic transgene delivery and the first description of systemically delivered *iNOS* for cancer gene therapy. Both *iNOS* gene therapy constructs provoked inhibition of clonogenicity in vitro. ·NO exerts its anti-cancer benefit when its intracellular concentration is in the micromolar range.^25^ Although we did not assess intracellular ·NO concentration following transfection, the accumulation of nitrites in the culture medium is indicative of a considerable increase in intracellular ·NO content. The fate of transfected cells likely depends on the degree of ·NO production but could include apoptosis, attraction of macrophages, or toxicity because of a bystander effect.^26^

A concern associated with indiscriminate production of ·NO is the deleterious side effects that may manifest, such as hypotension. Numerous strategies have been employed to limit ·NO production to the disease site, including β-galactosidase-provoked release of ·NO/nitroxyl (HNO) from isopropylamine (IPA)/NO,^27^ or the nitroreductase-dependent release of ·NO from 1-(2-methylpiperidin-1-yl)diazen-1-ium-1,2-diolate.^28^ Likewise, RRx-001, which preferentially releases ·NO in a hypoxic environment, attenuated murine squamous cell carcinoma (SCC) VII xenograft growth and sensitized to fractionated radiotherapy, doubling the survival time of mice.^29^ Ligand targeting of nanoparticles is a common targeting strategy, with tumor-associated dysregulated expression of the receptors of transferrin, folic acid, epidermal growth factor, and hyaluronic acid being particularly popular.^30^ We have previously employed numerous transcriptional targeting strategies. Utilization of the prostate-specific membrane antigen (PSMA) promoter elicited *iNOS* transgene expression in prostate cancer lines but not in colon or breast carcinoma lines.^11^ We have also used inducible promoters to control *iNOS* expression. The WAF1/p21 promoter, whose activity is induced by radiation, when used to drive *iNOS* expression, evoked RIF-1 and HT29 tumor growth delay that exceeded that observed with either a fractionated radiotherapy strategy alone^12^ or with a single X-ray dose (10 or 20 Gy).^14^

It was unsurprising that RALA/CMV-*iNOS* was more potent than RALA/hOC-*iNOS*, given the constitutive activity of the promoter, although the transcriptionally targeted therapy also significantly improved the survival of mice bearing metastases. Overexpression of *Runx2*, characteristic in MDA-MB-231,^31^ is responsible for activation of the hOC promoter.^18^ We have shown previously that PC3 prostate cancer cells express *GFP* and *iNOS* transgenes as provoked using hOC, but lymph node carcinoma of the prostate (LNCaP) cancer cells do not;^19^ LNCaP cells are known to express *Runx2* to a much lower extent than PC3 cells.^32^ Given that *Runx2* expression is elevated in metastatic bone lesions of breast cancer patients but absent in corresponding primary tumors,^31^ employment of a *Runx2*-activatable therapy should result in maximal *iNOS* transgene expression in the most aggressive tumor sites and spare normal tissue.^33^ *Runx2* has also been implicated in the progression of prostate,^34^ lung,^35^ and thyroid^36^ cancers, which preferentially target the bone for metastatic colonization. We expect that these and other tumors that overexpress *Runx2* would benefit from hOC-*iNOS* gene therapy.

Despite the compelling evidence of the therapeutic potential of *iNOS* gene therapy in neoplastic conditions,^9^, ^12^, ^14^, ^15^, ^16^, ^19^, ^37^, ^38^ the dichotomy of the relationship between ·NO and the tumor environment confers skepticism when it comes to overexpressing ·NO. Although *iNOS* expression was negatively correlated with lesion grade in a cohort of invasive ductal breast carcinomas,^39^ indicating a possible role of *iNOS* in the prevention of metastasis, *iNOS* expression has conversely been implicated as a marker of poor prognosis in several malignancies, including prostate, colon, and breast.^40^ Stratification of a breast cancer patient cohort by estrogen receptor (ER) expression revealed that *iNOS* expression was predictive of poorer survival in ER^−^ patients,^41^ whereas high *iNOS* expression was similarly detrimental in a range of triple-negative breast cancer patient cohorts.^42^ Consequently, efforts are being made to repress *iNOS* activity as a therapeutic strategy. NOS inhibitors such as aminoguanidine (AG)^22^ have been investigated in pre-clinical settings, and, more recently, ASP9853, an inhibitor of *iNOS* dimerization, was tested in combination with docetaxel in patients with advanced solid tumors.^43^

However, although *iNOS* expression may correlate with disease status in some analyses, it is important to note that protein levels do not necessarily correlate with activity. Several factors could affect the translation of *iNOS* mRNA to functional protein and the production of ·NO. In the mouse renal cancer (RENCA) cell line, *iNOS* mRNA expression is not translated into functional protein, resultant from post-transcriptional modification by microRNA (miR)-146a. Treatment of RENCA cells with anti-miR-146a restores the cells’ ability to translate iNOS protein with concurrent ·NO production, and xenografts of these cells had considerably slower growth dynamics than negative control anti-miR-treated cells.^44^ miR-146a expression may affect *iNOS* expression in the clinical setting, potentially complicating prognostication based on *iNOS* mRNA expression profiling. Indeed, miR-146a was overexpressed in triple-negative breast cancer cell lines (including MDA-MB-231) and was significantly overexpressed in triple-negative breast cancer patient samples compared with non-triple-negative patients.^45^ Another factor that plays a role in *iNOS* activity is its co-factor tetrahydrobiopterin. NOS enzymes in cancer cells may preferentially produce superoxide and peroxynitrite over ·NO itself, resultant from inappropriate tetrahydrobiopterin:dihydrobiopterin (BH4:BH2) proportions. Restoration of appropriate BH4:BH2 proportions in MCF-7 and MDA-MB-231 breast cancer cells using sepiapterin manifested a dose-dependent cytotoxicity that was diminished when NOS was inhibited. Oral sepiapterin also delayed MDA-MB-231 xenograft progression. In this model, aberrant BH4:BH2 proportion is likely to deprive the tumor of the therapeutic benefit afforded by ·NO.^46^

We investigated the effect of *iNOS* overexpression on sensitivity to docetaxel. A taxane, docetaxel acts by preventing microtubule depolymerization, inhibiting mitosis. We did not determine whether the additive effect we observed was due to sensitization to docetaxel or the additive effect of *iNOS* overexpression and docetaxel treatment. MDA-MB-231 cells treated with 100 nM docetaxel arrested in G2/M phase of the cell cycle,^47^ whereas treatment with the ·NO donor DETA-NONOate arrested MDA-MB-231 cells in G1.^48^ There is precedent for ·NO sensitizing to chemotherapy. In MDA-MB-231, hypoxia-induced resistance to doxorubicin and 5-hydroxytryptamine (5-HT) was attenuated by treatment with nitroglycerin (an ·NO donor). Low oxygen levels under conditions of hypoxia prohibit the biogenesis of ·NO, so these findings support a role for endogenous ·NO in chemosensitzation.^49^ CMV-*iNOS* treatment sensitized human cancer cells to cisplatin in vitro and also RIF-1 murine xenografts to the same in vivo.^9^ In a C6 glioma model, overexpression of dimethylarginine dimethylaminohydrolase (DDAH) (which metabolizes asymmetric dimethylarginine [ADMA], an endogenous NOS inhibitor) sensitized C6 xenografts to docetaxel.^50^ Additionally, in lung adenocarcinoma patients, nitroglycerin patch treatment improved the response to docetaxel/carboplatin therapy.^51^ It is likely that there is potential for RALA/*iNOS* therapy to similarly sensitize to docetaxel and that optimization of the regimen is required to determine the best therapeutic window in vivo. It is also possible that *iNOS* gene therapy may be of more benefit in a model of docetaxel resistance, which is more representative of those that have failed chemotherapy.

### Conclusions

Our data demonstrate a clear anti-cancer effect of RALA/*iNOS* gene therapy for metastatic breast cancer. Overexpression of *iNOS* with a concomitant increase in ·NO liberation is a strategy for direct cytotoxicity and requires additional interrogation for its ability to sensitize to other cytotoxic approaches. Measurement of circulating nitrites was a method for confirmation of *iNOS* transgene activity and could be harnessed to determine *iNOS* therapeutic efficacy. The nucleic acid delivery ability of RALA is unquestionable. Beyond utility as a reporter gene delivery vehicle,^1^ it effectively delivers small interfering RNAs (siRNAs),^2^ and RALA/DNA nanoparticles were evaluated as components of a DNA vaccination device.^21^ However, this is the first report validating systemically delivered RALA/nucleic acid therapeutics. Further development of this potent RALA/*iNOS* treatment is required with respect to dosing, adjuvant therapies, and increasing circulation times.

## Materials and Methods

### Materials

Unless otherwise stated, the reagents used were from Sigma.

### Cell Culture

ZR-75-1 breast cancer cells were purchased from the ATCC and maintained in RPMI 1640 medium (Life Technologies) supplemented with 10% FBS (PAA Laboratories). MDA-MB-231-luc-D3H1 cells were purchased from PerkinElmer and maintained in DMEM (Life Technologies) supplemented with 10% FBS (PAA). Cells were cultivated in 175-cm^2^ flasks in a humidified incubator. When 80%–90% confluency was reached, cells were passed to maintain exponential growth. Mycoplasma absence was confirmed monthly using Plasmotest (InvivoGen).

### Plasmid DNA Preparation

MAX Efficiency DH5α-competent cells containing relevant plasmids (pEGFP-N1/CMV-*iNOS*/hOC-*iNOS*) were cultured in a shaking incubator overnight at 37°C in Luria broth containing the appropriate antibiotic. Plasmid DNA was isolated and purified using PureLink HiPure Plasmid Maxiprep Kits (Life Technologies) using the manufacturer’s protocol. Plasmid DNA was dissolved in ultrapure water and stored at −20°C.

### Nanoparticle Complexation and Characterization

RALA was custom-synthesized using solid-state synthesis (fluorenylmethyloxycarbonyl [FMOC]) (Biomatik) and supplied as a desalted lyophilized powder. Reconstitution was in ultrapure water to a stock concentration of 5.8 mg/mL. Aliquots were stored at −20°C until use.

Plasmid DNA/RALA nanocomplexes were constructed as described previously.^1^ Briefly, plasmid DNA was incubated with RALA for 30 min at room temperature to facilitate electrostatic interaction of the anionic DNA with the cationic peptide. Nanoparticles were complexed at N:P10 (the N:P ratio is the molar ratio of positively charged nitrogen atoms in the peptide to negatively charged phosphates in the pDNA backbone—at N:P10, 14.5 μg of RALA is used to neutralize 1 μg of DNA). Nanoparticles were analyzed in terms of their hydrodynamic size and particle charge using a Nano ZS Zetasizer and DTS software (Malvern Instruments).

### Intracellular Nanoparticle Tracking

Plasmid DNA (pEGFP-1, analogous to pEGFP-N1 but lacking the promoter) was labeled with Cy3 using a Mirus Bio LabelIt kit (Cambridge Bioscience) as recommended by the manufacturer. Cy3-labeled DNA was complexed with RALA as before, and the effect of the fluorophore on nanoparticle size and charge was determined as above.

MDA-MB-231-luc-D3H1 cells were seeded in 24-well plates on round coverslips at 10^4^ cells/coverslip and allowed to adhere for 2 hr. The wells were then supplemented with complete growth medium and incubated overnight. Following 2 hr of starvation in Opti-MEM (Life Technologies), nanoparticle complexes were added to the Opti-MEM, and cells were transfected for 30, 60, and 120 min. The cell actin cytoskeleton was stained using fluorescein isothiocyanate (FITC)-conjugated phalloidin (Life Technologies), and coverslips were mounted onto microscope slides using Diamond Antifade with DAPI (Life Technologies).

Nanoparticle subcellular localization was analyzed in MDA-MB-231-luc-D3H1 cells by confocal fluorescence microscopy using a Leica SP5 microscope and LAS-AF software.

### Clonogenic Assay

The effect of RALA/*iNOS* on the clonogenicity of MDA-MB-231-luc-D3H1 cells was assessed. MDA-MB-231-luc-D3H1 cells were seeded in T25 culture flasks at a density of 10^6^ cells/flask and incubated overnight. Following 2-hr starvation in Opti-MEM, cells were transfected with RALA/CMV-*iNOS* or RALA/hOC-*iNOS* nanoparticle formulations, equivalent to 6 μg DNA per flask. Transfection was for 6 hr, and then transfection media were replaced with normal growth medium, and cells were returned to the incubator for overnight incubation. After 24 hr, cells were trypsinized, counted, and plated in triplicate in 6-well plates at 500 or 1,000 cells/well. Plates were incubated at 37°C for 12 days, and then colonies were fixed and stained using 0.4% crystal violet (Sigma) in 70% methanol. Excess stain was removed by gentle washing in water, and when dry, colonies were manually counted. Treatment with 1 mM aminoguanidine (a NOS inhibitor), where appropriate, began 24 hr after plating into clonogenic plates.

### Vector Neutralization Assay

Before commencing in vivo therapeutic assessment of RALA/*iNOS* nanoparticles, we determined whether repeated administration of nanoparticles induced vector neutralization in a competent immune system. Nanoparticles (comprising 10 μg pEGFP-N1 complexed with RALA at N:P10) were formulated as above in a volume of 100 μL. Treatments were delivered via the tail vein of male C57BL/6 mice (6–8 weeks old at the beginning of the experiment) using a 29G insulin syringe (Terumo). PBS and DNA- and RALA-only treatments were also performed. Treatments were administered once, twice, or three times (for multiple administrations, 1 week elapsed between treatments). 1 week after final administration, mice were sacrificed by CO_2_ asphyxiation. Blood was collected by cardiac puncture, serum was isolated, and sera from triplicate mice were pooled, heat-inactivated, and stored at −20°C.

5 × 10^3^ ZR-75-1 cells were seeded in triplicate wells of 96-well plates and allowed to adhere overnight. Cells were starved in Opti-MEM for 2 hr prior to transfection. RALA/pEGFP-N1 nanoparticles were prepared and incubated for 30 min in Opti-MEM containing sera (0%, 0.1%, 1%, and 10% serum) from mice that had received the indicated treatment. Transfections were for 6 hr, and then Opti-MEM was replaced with RPMI 1640 medium. 48 hr later, cells were analyzed for *eGFP* expression by fluorescence microscopy using a Nikon Eclipse TE300 fluorescence microscope and by flow cytometry using a Becton Dickinson FACSCalibur.

### Neutralizing Antibody Assay

Serum-neutralizing antibody content was analyzed by ELISA.^52^ Maxisorp ELISA plates (Nunc) were coated overnight at 4°C with RALA/pEGFP-N1 nanoparticles in PBS. Wells were washed with PBS/0.05% Tween 20 and blocked with PBS/1% BSA for 1 hr at room temperature. The wells were probed (1 hr, room temperature) with diluted sera (1:10, 1:100, and 1:1,000) from mice that had received PBS, pEGFP-N1, RALA, or RALA/pEGFP-N1 nanoparticles, washed three times with PBS/0.05% Tween 20, and probed with an anti-mouse immunoglobulin A (IgA),M,G-horseradish peroxidase (HRP) secondary antibody (AdB Serotec). Following three further washes, tetramethylbenzidine substrate was added, quenched with 1 N HCl, and absorbance was quantified at 450 nm, with background absorbance (550 nm) subtracted.

### *iNOS* Transgene Expression

MDA-MB-231-luc-D3H1 cells were plated (10^4^ cells/well of a 24-well plate), allowed to adhere overnight, and transfected with RALA/CMV-*iNOS* or RALA/hOC-*iNOS* for 6 hr, and then Opti-MEM was replaced with phenol red-free MEM/10% FBS. 48 hr later, MEM nitrite content was assayed using Greiss test for nitrites (Active Motif) as instructed by the manufacturer. Cellular *iNOS* transgene expression was measured via western blot as described previously.^11^

### *iNOS*-Mediated Docetaxel Sensitization

MDA-MB-231-luc-D3H1 cells were plated in 24 well plates at 10^5^ cells/well and allowed to attach overnight. Cells were transfected with RALA/hOC-*iNOS* or RALA/CMV-*iNOS* nanoparticles (0.5 μg DNA/well) for 6 hr, and then cells were returned to DMEM. 24 hr following transfection, DMEM was replaced with DMEM containing docetaxel (0, 4, 20, 100, 500, and 2,500 ng/mL). Following a further 48-hr incubation, docetaxel-containing DMEM was replaced with DMEM containing D-luciferin (PerkinElmer) at 150 μg/mL. Subsequent to a 2-min incubation, luminescence was determined using IVIS imaging. Luminescence in wells was quantified using Living Image software (PerkinElmer).

### Establishment of Metastatic Disease

All animal experiments were carried out in accordance with the Animal (Scientific Procedures) Act of 1986 and conformed to the current United Kingdom Co-ordinating Committee on Cancer Research (UKCCCR) guidelines. Mice were bred in-house and maintained using the highest possible standard of care, and priority was given to their welfare.

Mice (6–8 weeks old) were anesthetized using isoflurane (3% in O_2_) and restrained in a supine position using surgical adhesive tape. Thoracic fur was removed. Using a 1-mL syringe fitted with a 26G needle, mice were implanted with 100 μL of MDA-MB-231-luc-D3H1 cells at 10^6^ cells/mL via the left cardiac ventricle. The cell suspension was gently injected into the ventricle, and then the needle was held in place for 10 s to minimize leakage of the delivered cells from the ventricle. To confirm appropriate delivery, mice were injected with 150 mg/kg D-luciferin intraperitoneally (i.p.), and, after 15-min incubation, isoflurane-anesthetized mice were imaged using an IVIS200 (Xenogen) imaging system. Appropriate left ventricular delivery was indicated by the appearance of luminescence throughout the mouse, whereas inappropriate delivery was indicated by luminescence being limited to the thoracic cavity; such mice were sacrificed by CO_2_ asphyxiation.

### Gene Therapy Regimen

Beginning 48 hr post-implantation, mice received treatments twice weekly for five treatments. Treatments comprised RALA/CMV-*iNOS* or RALA/hOC-*iNOS* complexes (corresponding to 5 × 10 μg DNA per mouse) at N:P10, whereas vehicle control (RALA equivalent to the mass of RALA used in the gene therapy regimens) and untreated controls were also performed. Treatment was via tail vein injection. A subgroup of mice received docetaxel treatment in addition to *iNOS* gene therapy. Docetaxel treatment (5 mg/kg i.p.) commenced 7 days post-implantation and was given weekly for 3 weeks; gene therapy treatments were as before.

Mice were monitored for micrometastasis development using routine (twice weekly) IVIS imaging as described above as well as body mass measurement. A loss of 20% of original body mass was deemed sufficient to necessitate sacrifice of the mouse. The degree of whole-body luminescence in mice was determined using Living Image software (PerkinElmer). At regular intervals, blood samples were taken from mice following a tail prick and stored in EDTA-coated tubes. Blood nitrite levels were assayed using the ArrowSTRAIGHT nitric oxide measurement system (Lazar Labs).

### Statistics

All statistics were performed using GraphPad Prism, version 6.0g for Mac OS X. The various tests used are described throughout.

## Author Contributions

Conceptualization, N.J.D., V.L.K., T.R., and H.O.M.; Methodology, C.M.M., J.W.M., J.M., A.A.A., and J.A.C.; Investigation, C.M.M., J.W.M., J.M., A.A.A., and J.A.C.; Writing – Original Draft, C.M.M. and H.O.M.; Writing – Review & Editing, C.M.M. and H.O.M.; Funding Acquisition, V.L.K., T.R., and H.O.M.; Resources, V.L.K., T.R., and H.O.M.; Supervision, V.L.K., T.R., and H.O.M.

## Conflicts of Interest

The authors declare that they have no conflicting interests.

## Supplemental Information

Document S1. Figures S1–S5

Document S2. Article plus Supplemental Information
