# Supplementary material for: Systemic RALA/iNOS Nanoparticles: A Potent Gene Therapy for Metastatic Breast Cancer Coupled as a Biomarker of Treatment
Source: Mol Ther Nucleic Acids. 2016 Dec 31;6:249–58. doi: 10.1016/j.omtn.2016.12.010 (PMC5363505; doi:10.1016/j.omtn.2016.12.010)
Supplement: Document S1. Figures S1–S5 [file mmc1.pdf]

OMTN, Volume *6*

Supplemental Information

Systemic RALA/iNOS Nanoparticles: A Potent Gene Therapy for Metastatic Breast Cancer Coupled as a Biomarker of Treatment

Cian M. McCrudden, John W. McBride, Joanne McCaffrey, Ahlam A. Ali, Nicholas J. Dunne, Vicky L. Kett, Jonathan A. Coulter, Tracy Robson, and Helen O. McCarthy

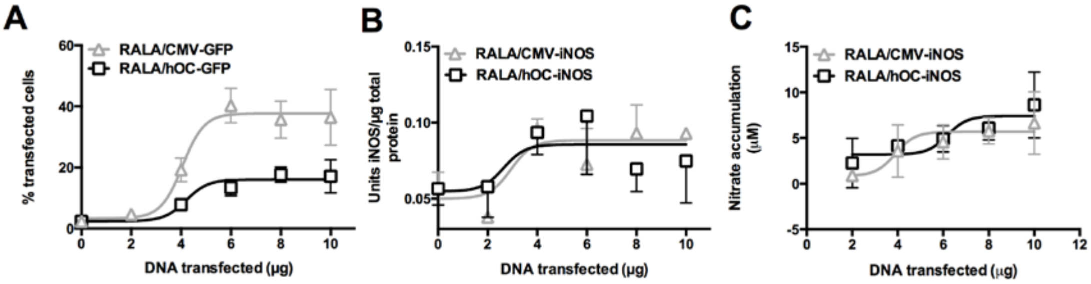

Figure S1 - Optimization of transfection with RALA/plasmid DNA constructs in MDA-MB-luc-D3H1. *A*, MDA-MB-luc-D3H1 Bioware® cells were transfected with pEGFP-N1 or hOC-GFP complexed with RALA at the indicated quantity of DNA per well (6-well plate). Transfection efficiency was assessed by flow cytometry. *B*, MDA-MB-luc-D3H1 Bioware® cells were transfected with RALA/CMV-iNOS or RALA/hOC-GFP; iNOS content was assessed by ELISA, and total protein content was determined by BCA assay to allow normalization of iNOS content. *C*, iNOS activity was assessed using the Greiss assay.

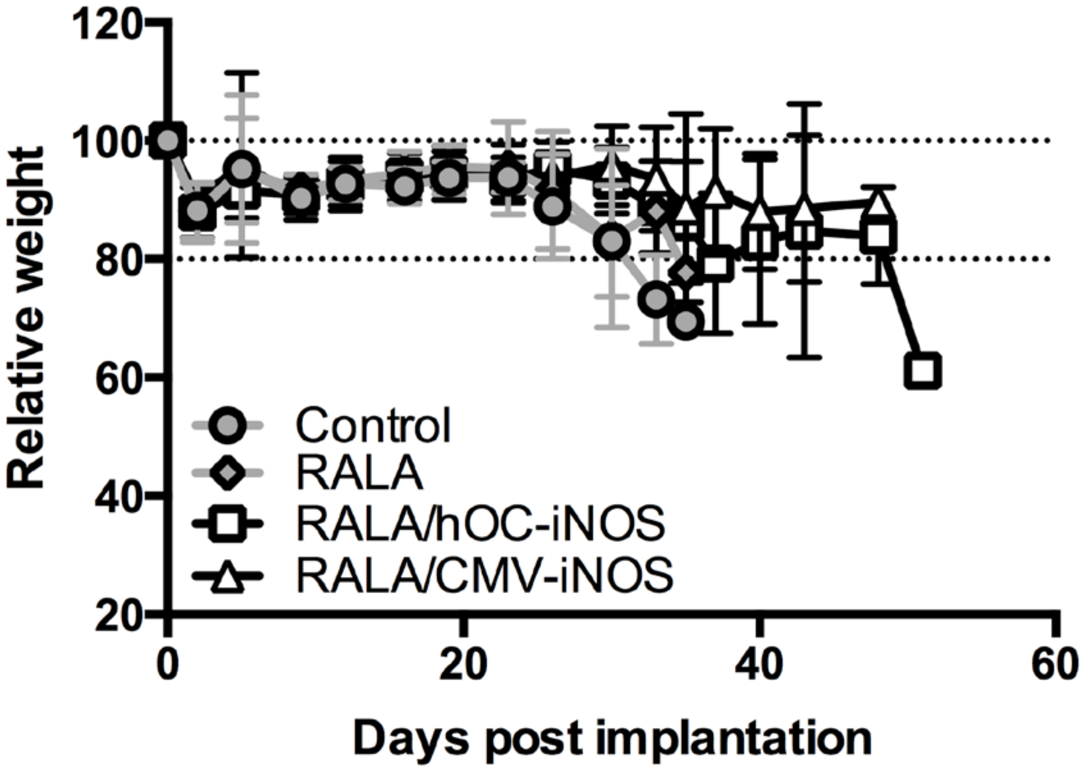

Figure S2 - Weight loss in all mice in gene therapy groups.

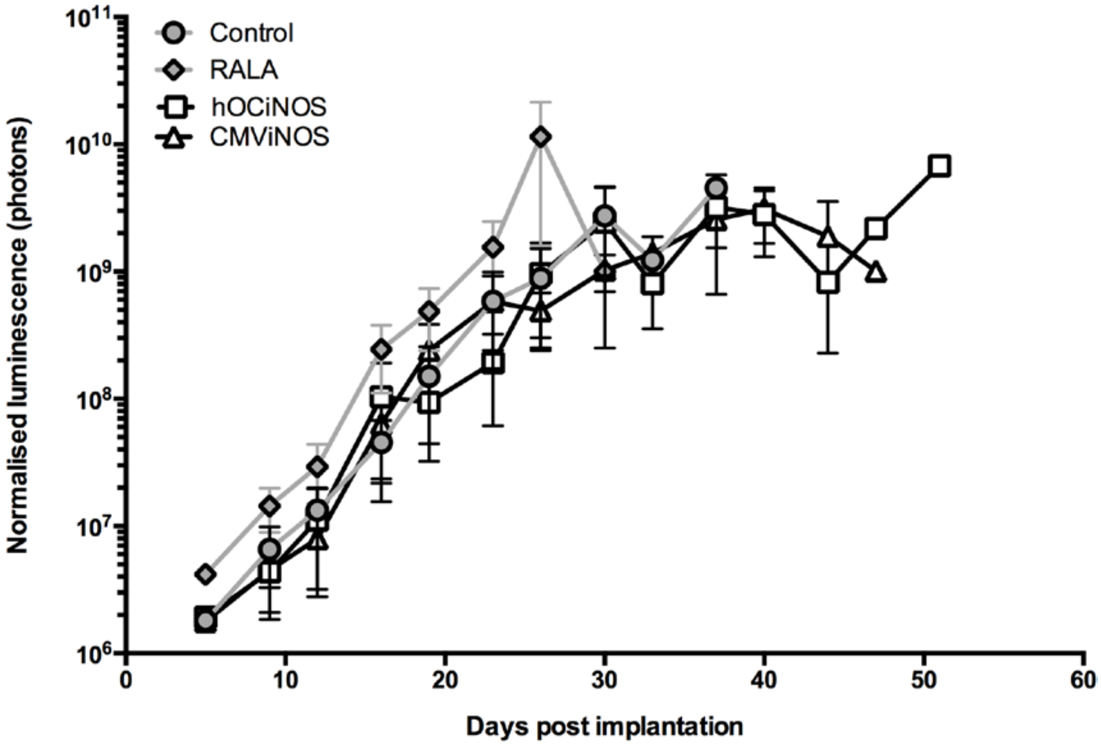

Figure S3 - Bioluminescence accumulation in mice in gene therapy groups.

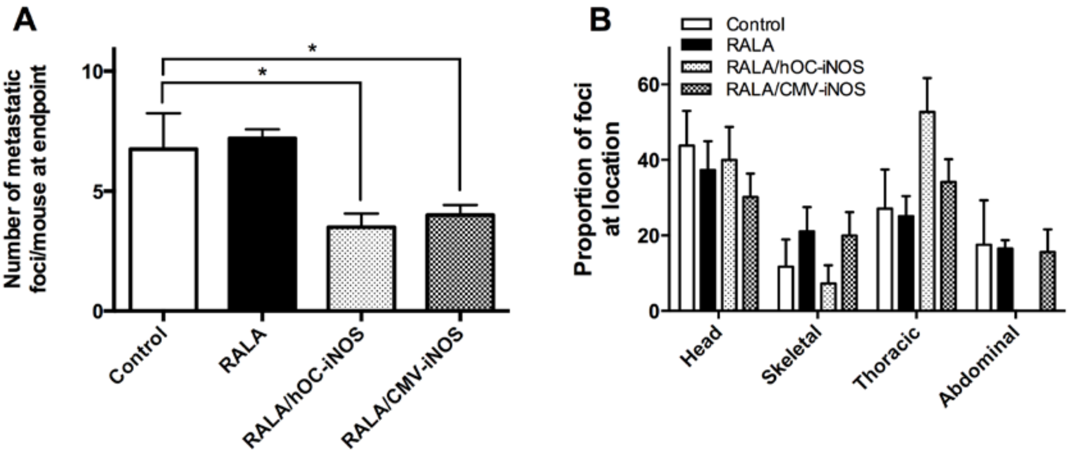

Figure S4 - Number and location of metastatic foci per mouse.

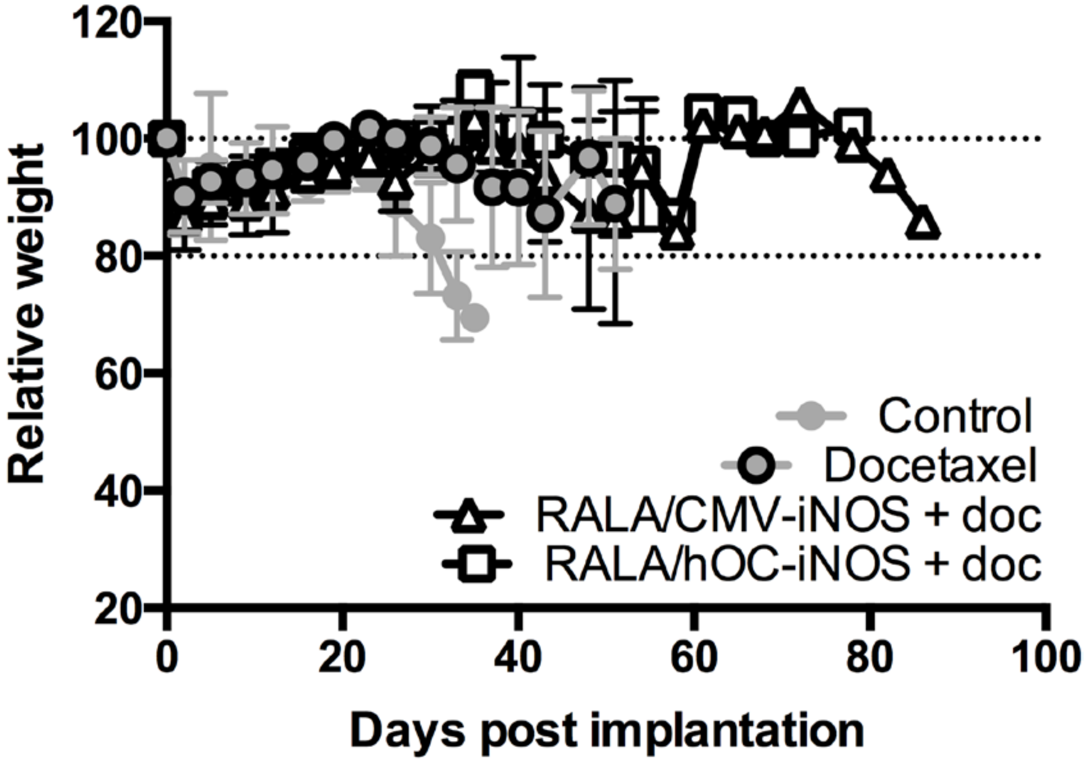

Figure S5 - Weight loss in all mice in docetaxel +/- gene therapy groups.
